# Development and evaluation of a nomogram for adverse outcomes of preeclampsia in Chinese pregnant women

**DOI:** 10.1186/s12884-022-04820-x

**Published:** 2022-06-20

**Authors:** Jiangyuan Zheng, Li Zhang, Yang Zhou, Lin Xu, Zuyue Zhang, Yaling Luo

**Affiliations:** 1 College of Medical Informatics, Chongqing Medical University, Chongqing, China; 2 College of Nursing, Chongqing Medical University, Chongqing, China; 3 Medical Data Science Academy, Chongqing Medical University, Chongqing, China

**Keywords:** Preeclampsia, Prediction, Adverse pregnancy outcomes, Nomogram

## Abstract

**Objective:**

Preeclampsia, the main cause of maternal and perinatal deaths, is associated with several maternal complications and adverse perinatal outcomes. Some prediction models are uesd to evaluate adverse pregnancy outcomes. However, some of the current prediction models are mainly carried out in developed countries, and many problems are still exist. We, thus, developed and validated a nomogram to predict the risk of adverse pregnancy outcomes of preeclampsia in Chinese pregnant women.

**Methods:**

The clinical data of 720 pregnant women with preeclampsia in seven medical institutions in Chongqing from January 1, 2010, to December 31, 2020, were analyzed retrospectively. The patients were divided into two groups: 180 cases (25%) with adverse outcomes and 540 cases (75%) without adverse outcomes. The indicators were identified via univariate analysis. Logistic regression analysis was used to establish the prediction model, which was displayed by a nomogram. The performance of the nomogram was evaluated in terms of the area under the receiver operating characteristic (ROC) curve, calibration, and clinical utility.

**Results:**

Univariate analysis showed that 24 indicators were significantly different (P < 0.05). Logistic regression analysis showed that gestational age, 24 h urine protein qualitative, and TT were significantly different (P < 0.05). The area under the ROC curve was 0.781 (95% CI 0.737–0.825) in training set and 0.777 (95% CI 0.689–0.865) in test set. The calibration curve of the nomogram showed good agreement between prediction and observation. The analysis of the clinical decision curve showed that the nomogram is of practical significance.

**Conclusion:**

Our study identified gestational age, 24 h urine protein qualitative, and TT as risk factors for adverse outcomes of preeclampsia in pregnant women, and constructed a nomogram that can easily predict and evaluate the risk of adverse pregnancy outcomes in women with preeclampsia.

## Introduction

Preeclampsia is a pregnancy-specific disease characterized by hypertension after 20 weeks of gestation, with or without urinary protein, or with impaired noble organ function [1]. Preeclampsia is the main cause of maternal and perinatal mortality, posing a serious threat to maternal and child health and even life itself [2, 3]. In the past few decades, a large number of scholars at home and abroad made significant progress in the field of preeclampsia; however, to date, the etiology and pathogenesis of preeclampsia are not fully explained [4, 5]. At present, domestic and foreign scholars generally believe that the main pathogenesis of preeclampsia is placental malformation and insufficient blood supply, which result in the release of inflammatory factors and cell debris into the blood, causing maternal systemic inflammatory response and endothelial dysfunction [6–8]. Preeclampsia has no effective treatment, and the only solution is to terminate the pregnancy [9, 10].

Preeclampsia affects approximately 2%–8% of pregnant women globally each year [11], with a higher incidence in developing countries than in developed countries [12]. With the development of the social economy and the adjustment of the fertility policy in China, older women are more likely to give birth again, increasing the proportion of older mothers and the prevalence of preeclampsia [13]. Studies showed that [14–16] after the age of 35 years, women’s fertility and physical function begin to decline gradually, which is influenced by several risk factors in the environment and society for a long time, and the risk of obstetric complications and perinatal adverse outcomes of older mothers is increased. The number of older pregnant women increases year by year, which has become a global problem. Moreover, the problem of older pregnant women is particularly prominent due to the adjustment of the fertility policy in China [17–19].

Some biomarkers (such as sFlt-1 and PlGF) are reported to be useful in the early diagnosis of preeclampsia and in facilitating the prediction of maternal–fetal outcomes [20–22]. In recent years, scholars at home and abroad use several preeclampsia examination indicators to build a prediction model to predict pregnant women with preeclampsia and severe maternal outcomes and achieved good results [23–27]. However, most of these studies were conducted in developed countries, with predominantly Caucasian subjects and relatively few East Asian subjects. Additionally, there are still some problems with the established prediction models, such as excessive indicators in models, which makes the equation complex and inconvenient in clinical application. Furthermore, some indicators are costly, making the application difficult to promote in low- and middle-income countries [28]. Moreover, most of the models were not validated externally neither were they validated by decision curve analysis. Only the area under the ROC curve was used to evaluate the models, and the net benefit and clinical utility of the predictive models were not evaluated.

Because of the imbalance in the quantity and quality of medical resources in different regions of China, primary healthcare institutions and primary healthcare workers urgently need early prediction tools to screen high-risk pregnant women with preeclampsia and timely achieve referral to improve the adverse pregnancy outcomes of mothers and infants. Therefore, we established a multivariate prediction model of adverse pregnancy outcome in preeclampsia by collecting the indicators of routine prenatal care of pregnant women with preeclampsia in several hospitals; drawing the nomogram; and using the area under the ROC curve, calibration chart, and clinical decision curve to evaluate the prediction model.

## Materials and methods

### Study population

This study was a retrospective study performed in the big data platform which belonged to Chongqing Medical University Medical Data Science Academy. The platform contained the Chongqing Medical University affiliated 7 medical institutions of electronic case data. We collected the information of pregnant women who were diagnosed with preeclampsia from January 1, 2010, to December 31, 2020, including their baseline data, laboratory test results, maternal complications, and fetal pregnancy outcomes. Pregnant women with a diagnosis of chronic hypertension with preeclampsia, women in the state of pregnancy, and pregnant women with much missing clinical data were excluded. Finally, 720 pregnant women with preeclampsia were included. The patients were divided into two groups according to the adverse outcomes: the group with adverse outcomes (*n* = 180) and the group without adverse outcomes (*n* = 540). The research proposal was approved by the Ethics Committee of Chongqing Medical University.

### Diagnostic criteria

According to Diagnosis and treatment of hypertension and pre-eclampsia in pregnancy: a clinical practice guideline in China(2020) [29]: the diagnostic criteria of mild preeclampsia were systolic blood pressure ≥ 140 mmHg and/or diastolic blood pressure ≥ 90 mmHg with urine protein ≥ 0.3 g/24 h or random urine protein ≥ ( +) after 20 weeks of pregnancy. The diagnostic criteria for severe preeclampsia were as follows: ① A continuous increase in blood pressure: systolic pressure ≥ 160 mmHg and/or diastolic pressure ≥ 110 mmHg. ② Urinary protein ≥ 2.0 g/24 h or random urinary protein ≥ (+ +). ③ Serum creatinine ≥ 1.2 mg/dL (unless it was known to be elevated before). ④ Platelet count < 100,000/ml (< 100 × 10^9^/L). ⑤ Microangiopathic hemolysis: LDH increased. ⑥ Elevation of serum transaminases. ⑦ Persistent headache or other cerebral or visual disturbances. ⑧ Persistent epigastric pain.

### Clinical data collection

The risk factors included in the study variables were as follows: ① Basic information: age, body mass index (BMI), fertility, prenatal examination, in vitro fertilization and embryo transfer (IVF-ET), intrahepatic cholestasis of pregnancy (ICP), gestational diabetes mellitus (GDM), cardiovascular system disease, immune system disease, etc. ② Symptoms and signs on admission: edema, expiratory dyspnea, dizziness, headache, blurred vision, increased blood pressure, increased pulse, increased respiratory rate, etc. ③ Laboratory test index: 24 h urine protein qualitative, liver and kidney function indexes, coagulation index, etc.

The primary outcome of this study was adverse pregnancy outcome in patients with preeclampsia, including the following maternal outcomes: placental abruption, neonatal asphyxia, intrauterine fetal distress, fetal growth restriction, low birth weight infants, preterm delivery (less than 37 weeks of gestation). In addition, Age ≥ 35 was defined as advanced maternal age [30]. Gestational age < 34 was defined as early-onset preeclampsia and gestational age between 34 and 37 was defined as late-onset preeclampsia, whereas gestational age ≥ 37 was defined as full-term preeclampsia[31, 32]. Low birth weight was defined as < 2500 g [33]. Fetal growth restriction was defined as an estimated fetal weight less than the 10th percentile for gestational age [34].

### Statistical analysis

Missing data were filled with missForest. SPSS 25.0 was used for univariate analysis of risk factors for adverse outcomes of preeclampsia in pregnant women. Skewed continuous data were presented as median values and interquartile ranges and analyzed using the Mann–Whitney U test, whereas categorical data were presented as frequencies and proportions and analyzed using the chi-square test. Statistically significant factors during the univariate analysis were entered into the multivariate logistic regression analysis, using the stepwise forward method. The “rms” package in R was used for plotting nomograms and calibration curves. The internal validation of the model was evaluated using the area under the ROC curve. The clinical utility of nomograms was determined by quantifying the net benefit at different threshold probabilities in the dataset. All statistical tests were two-sided, and *P* values < 0.05 were considered statistically significant.

## Results

**Fig. 1:**
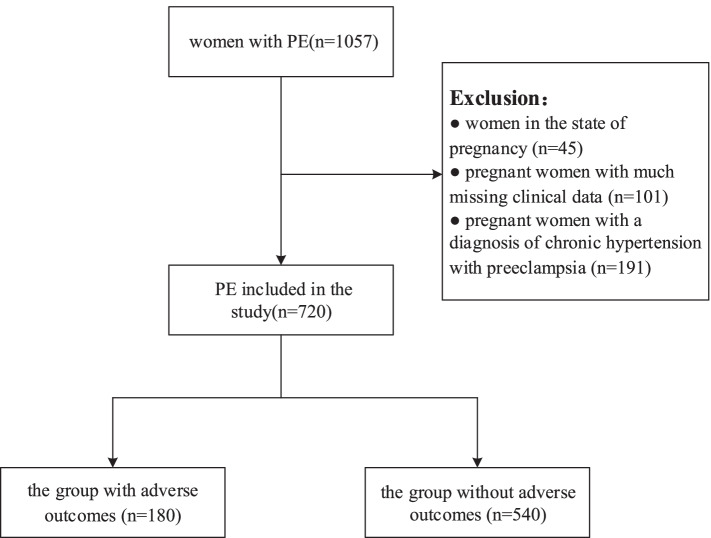
Flowchart of this study

In this study, 180 patients have adverse pregnancy outcomes and 540 patients have no adverse pregnancy outcomes (Fig. 1). The single-factor test and the chi-square test were used to analyze the indicators included in the study. The significantly associated indicators were as follows: gestational age, type of operation, 24 h urine protein qualitative, edema, nausea and vomiting, dizziness and headache, blurred vision, fundus disease, ICP, SBP, DBP, BMI, ALT, AST, TT, APTT, BUC, ALB, Cr, HB, WBC, TBIL, DBIL, and IBIL (Tables 1 and 2).

**Table 1:** Univariate analysis of adverse outcomes in preeclampsia (chi-square test)

Clinical Index	Control Group *n* = 540	Study Group *n* = 180	*χ*^*2*^	*P*
Age			0.014	0.907
< 35	452(83.70%)	150(83.30%)		
≥ 35	88(16.30%)	30(16.70%)		
Gestational age			134.815	< 0.001***
< 34	28(5.20%)	59(32.70%)		
34–37	113(20.90%)	64(35.60%)		
≥ 37	399(73.90%)	57(31.70%)		
Primipara	333(61.70%)	103(57.20%)	1.116	0.291
Type of operation			7.475	0.006**
Natural labor	74(13.70%)	11(6.10%)		
Cesarean section	466(86.30%)	169(93.90%)		
IVF-ET	23(4.30%)	6(3.30%)	0.299	0.584
Twins	17(3.10%)	9(5.00%)	1.33	0.249
24 h urine protein qualitative			50.25	< 0.001***
+	189(35.10%)	34(18.80%)		
+ +	206(38.10%)	46(25.60%)		
+ + +	139(25.70%)	95(52.80%)		
+ + + +	6(1.10%)	5(2.80%)		
Periodic antenatal examination	422(78.10%)	132(73.30%)	1.764	0.184
Edema	182(33.70%)	82(45.60%)	8.166	0.004**
Pain and chest distress	2(0.40%)	2(1.10%)	1.341	0.247
Expiratory dyspnea	1(0.20%)	1(0.60%)	0.669	0.414
Nausea and vomiting	1(0.20%)	3(1.70%)	5.363	0.021*
Dizziness and headache	25(4.60%)	20(11.10%)	9.679	0.002**
Blurred vision	18(3.30%)	13(7.20%)	4.955	0.026*
Respiratory disease	2(0.40%)	0(0.00%)	0.669	0.414
Cardiovascular disease	12(2.20%)	7(3.90%)	1.46	0.227
GDM	133(24.60%)	33(18.30%)	3.017	0.082
Immune system disease	3(0.60%)	1(0.60%)	0	1
Fundus disease	13(2.40%)	12(6.70%)	7.307	0.007**
Thyroid disease	58(10.70%)	16(8.90%)	0.502	0.479
ICP	34(6.30%)	22(12.20%)	6.609	0.01*

(Significance levels: ‘***’0.001, ‘**’0.01, ‘*’0.05.)

**Table 2:** Univariate analysis of adverse outcomes in preeclampsia (Mann–Whitney U test)

Clinical Index	Control Group *n* = 540, M (IQR)	Study Group *n* = 180, M (IQR)	*Z*	*P*
SBP, mmHg	142(136,152.99)	149(138,160)	-3.733	< 0.001***
DBP, mmHg	93.09(88,100)	97.5(88.25,106)	-2.829	0.005**
Pulse, times/min	87(80,95.15)	88(80,96)	-0.111	0.912
Respiratory Frequency, times/min	20(19.74,20)	20(19.94,20)	-1.546	0.122
BMI, kg/m^2^	28.79(27.47,30.25)	28.52(26.91,29.61)	-2.457	0.014*
ALT, U/L	15(10,26.08)	19(12.22,35.08)	-3.74	< 0.001***
AST, U/L	22.46(17.21,31.55)	27(21,39.51)	-4.48	< 0.001***
PT, s	10.91(10.4,11.6)	11.15(10.33,11.7)	-1.005	0.315
TT, s	16.3(14.9,17.42)	17.1(16.2,18.2)	-5.573	< 0.001***
APTT, s	28.7(26.02,32.1)	32.15(27.39,35.25)	-5.569	< 0.001***
BUC, µmol/L	421.14(359.23,486.84)	442.7(381.2,515.9)	-2.69	0.007**
ALB, g/L	33.15(29.81,36)	30.46(27.4,33.79)	-5.773	< 0.001***
FIB, g/L	4.2(3.64,4.88)	4.19(3.56,4.79)	-0.084	0.933
Cr, μmol/L	56.87(47.92,66.38)	61.61(51.9,76.3)	-3.943	< 0.001***
PLT, × 10^9^/L	170.5(139,212.32)	166.81(131,202.25)	-1.826	0.068
HB, g/dl	120(110,130)	124.5(115,137)	-3.651	< 0.001***
WBC, × 10^9^/L	8.45(7.12,10.05)	9.28(7.61,10.96)	-3.501	< 0.001***
RBC, × 10^9^/L	4.06(3.8,4.37)	4.15(3.75,4.58)	-1.872	0.061
TBIL, μmol/L	7.29(5.9,9.9)	7.13(5.36,8.98)	-2.027	0.043*
DBIL, μmol/L	2(1.6,2.7)	1.91(1.2,2.75)	-2.108	0.035*
IBIL, μmol/L	5.35(4.1,7.3)	5.06(3.82,6.49)	-2.083	0.037*

(*SBP* Systolic blood pressure, *DBP* Diastolic blood pressure, *BMI* Body mass index, *ALT* Alanine transaminase, *AST* Glutamic oxaloacetic transaminase, *PT* Prothrombin time, *TT* Thrombin time, *APTT* Activated partial thromboplastin time, *BUC* Blood uric acid, *ALB* Albumin, *FIB* Fibrinogen, *Cr* Creatinine, *PLT* Blood platelets, *HB* Hemoglobin, *WBC* White blood cell, *RBC* Red blood cell, *TBIL* Total bilirubin, *DBIL* Direct bilirubin, *IBIL* Indirect bilirubin; Significance levels: ‘***’0.001, ‘**’0.01, ‘*’0.05.)

Using logistic regression analysis, we identified three predictors that were significantly associated with adverse pregnancy outcomes in preeclampsia, including short gestational age (*P* < 0.001), high level of qualitative 24 h proteinuria qualitative (*P* < 0.01), and high TT (*P* < 0.05), as shown in Table 3.

**Table 3:** Parameter estimation and test results of multivariate Logistic model for adverse outcome of preeclampsia

Clinical Index	*β*	*SE*	*OR*	95% CI	*P*
Gestational age	-1.172	0.136	0.31	0.237–0.405	< 0.001
24 h urine protein qualitative	0.327	0.123	1.387	1.089–1.765	0.008
TT	0.11	0.045	1.116	1.023–1.218	0.014

### Establishment of a risk warning model for adverse outcomes of preeclampsia

**Fig. 2:**
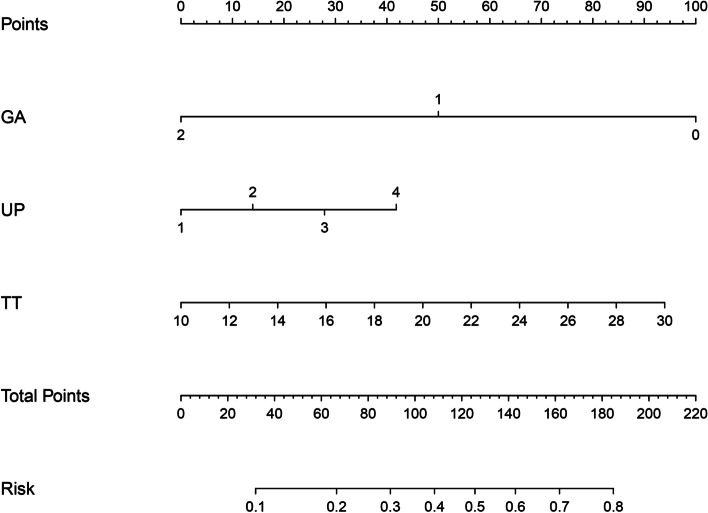
Nomogram. (Note: GA: Gestational age, UP: 24 h urine protein qualitative, TT: Thrombin time)

We established a predictive model for the adverse outcomes of preeclampsia based on the above independent predictors. For the convenience of clinical application and evaluation, we used R language to display the predictive model in the form of a nomogram (Fig. 2).

### Internal validation of the nomogram

**Fig. 3:**
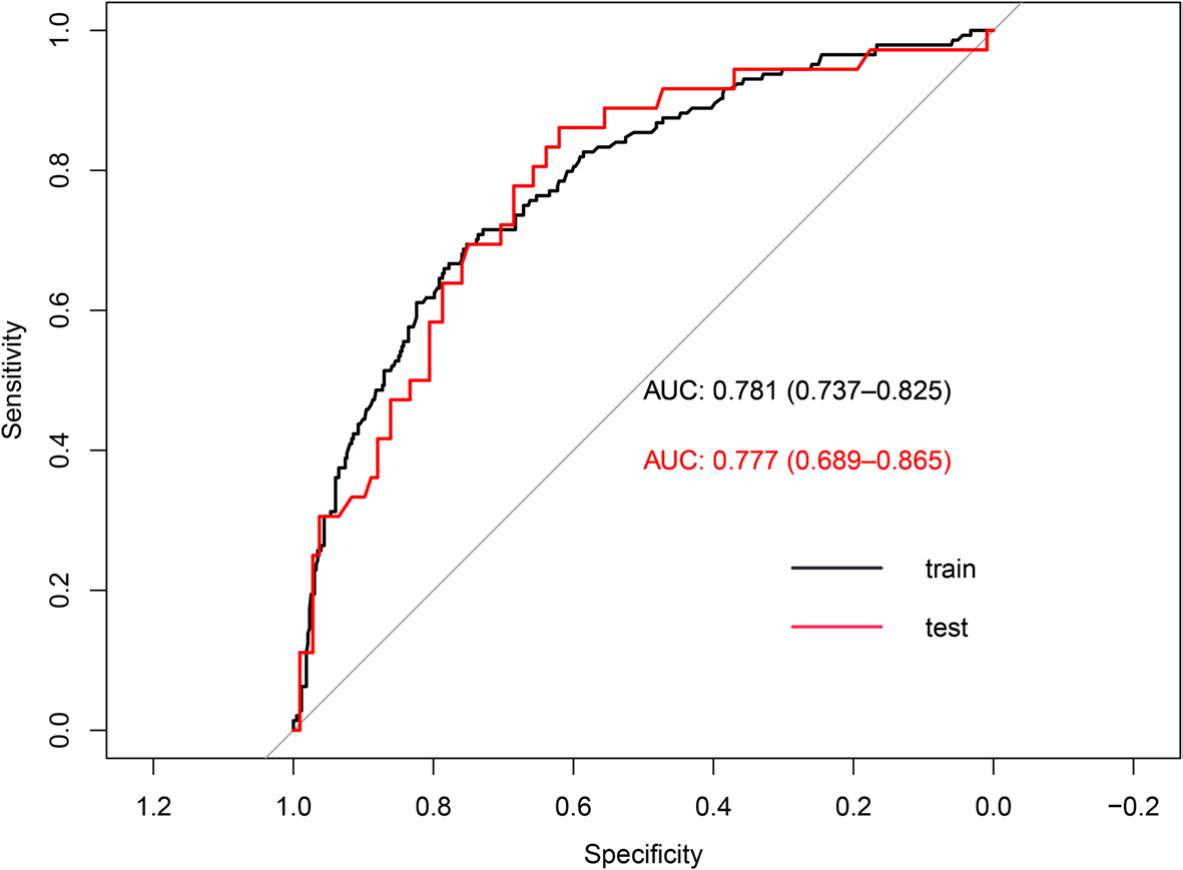
ROC of the nomogram

**Fig. 4:**
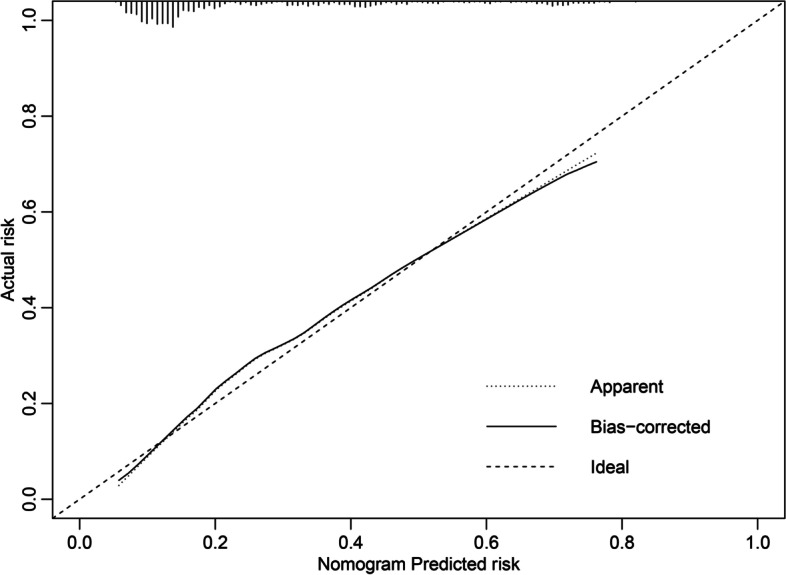
Calibration of the nomogram

A total of 720 cases of pregnant women with preeclampsia were randomly divided into 80% of the training set and 20% of the test set, and the ROC curves of the training set and the test set were drawn. The area under the ROC curve was 0.781 (95% CI 0.737–0.825) in the training set and 0.777 (95% CI 0.689–0.865) in the test set (Fig. 3). The calibration curve of the nomogram shows good agreement between predictions and observations (Fig. 4).

### Clinical practicability

**Fig. 5:**
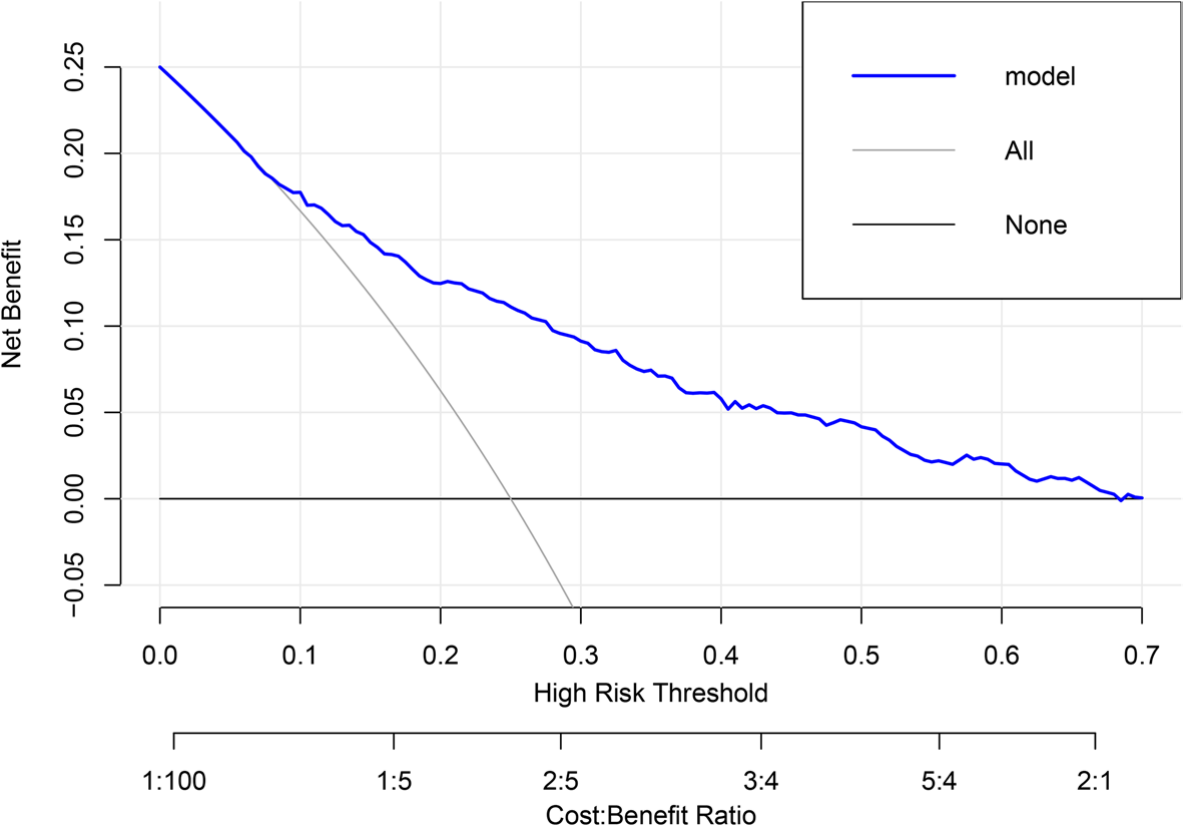
DCA of the nomogram

To assess the clinical utility of the model, we plotted a clinical decision curve (Fig. 5). The decision curve shows that the threshold probability of adverse outcomes of preeclampsia in pregnant women is in the range of 7%–70%, and if treatment measures are taken, the treatment of patients at such a time would have a net benefit.

## Discussion

In our study, 24 statistically significant factors were identified via univariate analysis, and the independent predictors of adverse pregnancy outcomes of preeclampsia were identified using the multivariate logistic stepwise forward regression method, including gestational age, 24 h urinary protein qualitative, and thrombin time; hence, the prediction model was established. Internal validation of the predictive model showed that the area under the ROC curve was 0.781 (95% CI 0.737–0.825) in the training set and 0.777 (95% CI 0.689–0.865) in the test set. From the prediction model of this study, we can see that if the gestational age of pregnant women with preeclampsia is < 34 weeks and there is an increase in 24 h urine protein qualitative and thrombin time, the risk of adverse outcomes would be higher. The model shows that the adverse outcomes of preeclampsia in pregnant women can be predicted by combining the general clinical characteristics, premonitory symptoms, and laboratory test results of pregnant women with preeclampsia.

Premature delivery is a common consequence of preeclampsia [35]. Premature birth is defined as a live birth at a gestational age < 37 weeks, when the neonatal organ systems are immature, which is one of the main reasons for the increased risk of perinatal and infant morbidity and mortality [36]. Preeclampsia occurring at a gestational age < 34 weeks was defined as early-onset preeclampsia whereas that occurring at a gestational age ≥ 34 weeks was defined as late-onset preeclampsia [37]. Several studies have reported that the incidence of adverse birth outcomes of early-onset preeclampsia is higher than that in late-onset preeclampsia, and the risk of multiple organ dysfunction is higher in early-onset preeclampsia than that in late-onset preeclampsia [38, 39]. Placental dysplasia is the main cause of early-onset preeclampsia. The abnormal invasion of trophoblast cells caused many pathophysiological changes in the inner wall of uterus, such as vascular reorganization and shallow implantation, which led to high resistance of blood vessels, insufficient perfusion of multiple organs and involvement of uterine spiral arteries[40]. Because of the decrease of placental blood supply and the aggravation of placental villus hypoxia and ischemia, the intake of nutrients and oxygen by the fetus decreases, which had adverse effects on the growth and development of the fetus. And it resulted in adverse outcomes such as FGR, fetal distress, hypoxia asphyxia and even death[41, 42]. Lisonkovas et al. [43] analyzed the clinical data of singleton pregnancies in 45,668 women and found that early-onset preeclampsia was associated with a significantly higher risk of adverse birth outcomes than late-onset preeclampsia. Belay Tolu et al. [44] also found that the rates of maternal and perinatal complications were higher in early-onset preeclampsia. Pregnant women with preeclampsia at < 34 weeks of gestation were at greater risk of adverse pregnancy outcomes in this study, which is consistent with the results of previous studies.

The morbidity or progression of preeclampsia is closely associated with proteinuria. Because of the systemic vascular endothelial injury and local ischemia, the obvious decrease of renal blood perfusion and glomerular filtration, the basement membrane is damaged and the permeability of blood vessels increased, which resulted in a large number of protein extravasation, and then preeclampsia patients had the symptoms of urinary protein[45]. In addition, the blood of preeclampsia patients was in a hypercoagulable state, the damage of renal tubular endothelial cells would lead to microthrombosis in blood vessels, which further aggravated the damage of renal function and increased the leakage of urinary protein[46]. We found that an increase in the qualitative level of 24 h urinary protein predicted a greater risk of adverse outcomes in patients, and this indicator was also included in the risk warning model. This is consistent with the results of previous studies [46, 47] that adverse maternal and neonatal outcomes are associated with the degree of increase in proteinuria. Studies showed that, the clinical signs and symptoms of preeclampsia appeared behind pathological basis changes[46, 47]. Due to the compensatory role of the kidney, urinary protein symptoms often appear later. When urinary protein occurred, the body might have hidden damage to multiple organ functions, including placenta and fetus. Thus, a progressive increase in albuminuria might indicate an exacerbation of the maternal condition and be associated with adverse fetal outcomes. With the study of the pathogenesis of preeclampsia, some scholars put forward some other points of view, that urine protein had nothing to do with the severity of preeclampsia. However, Henderson J T et al. [48] did not find a strong association between the degree of increased proteinuria and adverse outcomes of preeclampsia. Other articles [49, 50] also suggested that the severity of proteinuria could not be used to predict the risk of adverse pregnancy outcomes in patients with severe preeclampsia and that a ratio of urinary protein to creatinine that is > 0.3 could be used as a diagnostic indicator of preeclampsia; however, it could not be used to evaluate the deterioration of patients’ conditions and predict the prognosis of mothers and fetuses. Although the latest guidelines no longer regard urinary protein as a necessary condition for the diagnosis of preeclampsia, they cannot deny the significance of urinary protein in patients’ conditions.

Coagulation dysfunction has been reported to occur in patients with preeclampsia during the third trimester of pregnancy [51]. Previous studies [52, 53] showed that the blood of patients with preeclampsia is in a hypercoagulable state. As blood viscosity increases, pregnant women are prone to thrombosis. To get rid of these thrombus, the fibrinolytic system is activated [54], which consumes a large number of coagulation factors and platelets [55]. This results in coagulation function disorders that render pregnant women with preeclampsia more prone to postpartum hemorrhage, placental abruption, renal failure, heart failure, HELLP syndrome, and other complications, and may even lead to maternal and perinatal death in severe cases [56]. The thrombin time reflected whether there was sufficient well-formed fib in the plasma examined to meet the body's normal physiological clotting needs[57]. Our results showed that TT was higher in the study group than that in the control group. TT was also a risk factor for adverse pregnancy outcomes. There were two possible reasons: On the one hand, the endogenous coagulation factors and FIB synthesis in the liver of preeclampsia patients were insufficient due to hyperactivity of liver function and decrease of total plasma protein [52]. On the other hand, with the progress of preeclampsia, the continuous increase of blood pressure and the aggravation of vascular endothelial injury made the coagulation and anticoagulation pathways repeatedly activated, and the body in hypercoagulable state also might consume a large number of coagulation substances and enter a consumptive hypocoagulable state[58], thus causing a series of adverse pregnancy outcomes.

This study has several upsides. First, we drew a nomogram to visualize the model, which is easy to understand. At the same time, this study combines maternal factors and common prenatal laboratory data. Our prediction model contains only three variables, and compared with other models, the indicators are easy to identify and use. Second, we used internal validation, calibration curves, and clinical decision curve analysis to evaluate the efficacy and clinical utility of the model, which show good results in the above tests and reduce the bias caused by a single evaluation index to a certain extent. Third, our data come from multiple medical institutions, and the patients included are from multicenter surveys, with a good representative sample size.

Our research also has the following shortcomings: First, we performed a retrospective study due to the relatively low incidence of preeclampsia; thus, further prospective studies are needed to confirm our findings. Second, all the data in this study were collected from the Chongqing area; thus, there might be selection bias. Further external validation was needed to evaluate the performance of the model.

## Conclusions

In our study, we developed and validated a risk assessment model for adverse outcomes in Chinese pregnant women with preeclampsia. The model included three predictors: gestational age, 24 h proteinuria qualitative and TT. When a pregnant woman was diagnosed with preeclampsia, short gestational age, high level of 24 h proteinuria qualitative, and high TT might indicate serious adverse outcomes. The model is expected to be used as a decision support tool, especially in the areas where medical resources were scarce and in some primary hospitals in China. Clinicians can use the model to quantitatively assess the risk of preeclampsia patients and identify preeclampsia patients who may have adverse outcomes as soon as possible, so as to carry out intervention management and improve the outcome of mothers and infants.

## Data Availability

The datasets used during the current study can be obtained from the corresponding author on reasonable request.

## Abbreviations

ROC: Receiver operating characteristic
TT: Thrombin time
sFlt-1: Soluble vascular endothelial growth factor receptor-1
PlGF: Placental growth factor
LDH: Lactate dehydrogenase
BMI: Body mass index
IVF-ET: In vitro fertilization and embryo transfer
ICP: Intrahepatic cholestasis of pregnancy
GDM: Gestational diabetes mellitus
SBP: Systolic blood pressure
DBP: Diastolic blood pressure
ALT: Alanine transaminase
AST: Glutamic oxaloacetic transaminase
APTT: Activated partial thromboplastin time
BUC: Blood uric acid
ALB: Albumin
Cr: Creatinine
HB: Hemoglobin
WBC: White blood cell
TBIL: Total bilirubin
DBIL: Direct bilirubin
IBIL: Indirect bilirubin
PT: Prothrombin time
FIB: Fibrinogen
PLT: Blood platelets
RBC: Red blood cell
